# The Prospective Association of Dietary Sugar Intake in Adolescence With Risk Markers of Type 2 Diabetes in Young Adulthood

**DOI:** 10.3389/fnut.2020.615684

**Published:** 2021-01-18

**Authors:** Karen A. Della Corte, Katharina Penczynski, Gunter Kuhnle, Ines Perrar, Christian Herder, Michael Roden, Stefan A. Wudy, Thomas Remer, Ute Alexy, Anette E. Buyken

**Affiliations:** ^1^Public Health Nutrition, Paderborn University, Paderborn, Germany; ^2^Department of Food Safety, German Federal Institute for Risk Assessment (BfR), Berlin, Germany; ^3^Department of Food & Nutritional Sciences, Whiteknights, University of Reading, Reading, United Kingdom; ^4^DONALD Study, Nutritional Epidemiology, University of Bonn, Dortmund, Germany; ^5^Institute for Clinical Diabetology, German Diabetes Center, Leibniz Center for Diabetes Research at Heinrich Heine University, Düsseldorf, Germany; ^6^German Center for Diabetes Research (DZD), Oberschleissheim, Germany; ^7^Division of Endocrinology and Diabetology, Medical Faculty, Heinrich Heine University, Düsseldorf, Germany; ^8^Pediatric Endocrinology and Diabetology, Laboratory for Translational Hormone Analytics, Center of Child and Adolescent Medicine, Justus Liebig University Giessen, Giessen, Germany

**Keywords:** dietary sugar, urinary sugar, insulin sensitivity, fasting insulin, systemic inflammation

## Abstract

**Purpose:** To examine the prospective relevance of dietary sugar intake (based on dietary data as well as urinary excretion data) in adolescent years for insulin sensitivity and biomarkers of inflammation in young adulthood.

**Methods:** Overall 254 participants of the DONALD study who had at least two 3-day weighed dietary records for calculating intakes of fructose, glucose, sucrose, total, free, added sugars, total sugars from sugar-sweetened beverages (SSB), juice, and sweets/sugar or at least two complete 24 h urine samples (*n* = 221) for calculating sugar excretion (urinary fructose and urinary fructose + sucrose) in adolescence (females: 9–15 years, males: 10–16 years) and a fasting blood sample in adulthood (18–36 years), were included in multivariable linear regression analyses assessing their prospective associations with adult homeostasis model assessment insulin sensitivity (HOMA2-%S) and a pro-inflammatory score (based on CRP, IL-6, IL-18, leptin, chemerin, adiponectin).

**Results:** On the dietary intake level, no prospective associations were observed between adolescent fructose, sucrose, glucose, added, free, total sugar, or total sugar from SSB, juice or sweets/sugar intake and adult HOMA2-%S (*p* > 0.01). On the urinary level, however, higher excreted fructose levels were associated with improved adult HOMA2-%S (*p* = 0.008) among females only. No associations were observed between dietary or urinary sugars and the adult pro-inflammatory score (*p* > 0.01).

**Conclusion:** The present study did not provide support that dietary sugar consumed in adolescence is associated with adult insulin sensitivity. The one potential exception was the moderate dietary consumption of fructose, which showed a beneficial association with adult fasting insulin and insulin sensitivity.

## Introduction

It has been proposed that dietary sugar intake plays a causal role in the development of type 2 diabetes (T2D) (1–4), yet data on this topic are conflicting (5, 6). Due to its unregulated uptake and hepatic metabolism, the fructose component of high-sugar foods has been singled out as a key promotor of adverse cardiometabolic health outcomes when consumed in high amounts (7, 8). High intake levels of fructose administered in such intervention and acute studies do not however represent common intake patterns consumed habitually over time. In addition, dietary fructose that occurs naturally in whole fruits and vegetables provides only modest amounts of fructose combined with phytochemicals and fiber (9, 10), therefore amounts as well as types/sources of ingested fructose are of importance when considering its relation to risk factors of T2D (11). Dietary fructose elicits lower insulin secretion as compared to dietary glucose (12–14), and there is some evidence indicating that fructose intake/substitution can beneficially affect blood glucose levels (15, 16). Clarifications from prospective studies concerning the role of dietary fructose and other sugar types in the development of insulin sensitivity are needed.

It has additionally been postulated that dietary sugar intake leads to increased inflammatory processes in humans. While some evidence from human intervention trials points toward pro-inflammatory effects of sucrose and fructose vs. glucose (17, 18), our previous systematic review and meta-analysis of human intervention trials based on limited evidence found that dietary fructose does not contribute more to subclinical inflammation than other dietary sugars (19). Observational studies link the consumption of SSB to increased chronic inflammation (1, 20–22), yet it is unclear whether a modest and habitual sugar intake in adolescence is associated with later development of systemic inflammation.

Adolescents generally consume more added sugars (mainly as soft drinks) than other age groups (23, 24). Adolescence is also characterized by substantial hormonal, metabolic, and lifestyle changes, which is why this developmental stage is considered a critical period for later metabolic diseases (25). Dietary assessment methods are prone to measurement errors (26) and sugars are among the nutrients that are frequently underreported (27, 28) especially by adolescents who may be susceptible to socially desired reporting. Therefore, dietary biomarkers of 24 h urinary sucrose and urinary fructose have been introduced (29, 30), potentially allowing for greater accuracy in determining the impact dietary sugar intake during adolescence could have on adult metabolic health.

This analysis examined the prospective association between the intake of dietary sugar in adolescent years and the target outcomes of T2D risk factors (insulin sensitivity, fasting insulin, and systemic inflammation) measured in adulthood. By using a comprehensive approach, tests were performed on the basis of chemical sugar types (fructose, glucose, sucrose), sugar use (total sugar, added sugar, free sugar), and sugar sources [total sugars from sugar-sweetened beverage (SSB), juice, sweets/sugar] as well as urinary sugar excretion levels. This unique approach allows for a comprehensive investigation into how various forms of sugar measured on the self-reported dietary level as well as the biomarker level are related to risk factors for T2D.

## Materials and Methods

### Study Population

The present analysis was based on data from the DOrtmund Nutritional and Anthropometric Longitudinally Designed Study (DONALD Study), an open-ended and ongoing study conducted in Dortmund, Germany. In this cohort, approximately 35–40 healthy infants are recruited per year and first examined at the ages of 3 or 6 months. Each child returns for 3 more visits in the first year, 2 in the second year, and then once annually until adulthood. Between infancy and adulthood, detailed information on diet, metabolism, growth, and development are collected. This study began collecting this data in 1985. Components of the annual assessment and interview include anthropometric assessments, medical investigations, weighed 3-day dietary records and 24 h urine samples (from age 3–4 years onwards). Parental examinations (anthropometric measurements, lifestyle interviews) take place every 4 years. All examinations are performed with parental and later on, participants' written consent. Since 2005, participants are invited for follow-up in adulthood including fasting blood draw. The study has been previously described in more detail (31), and was approved by the Ethics Committee of the University of Bonn (Germany) according to the guidelines of the Declaration of Helsinki.

### Study Sample

At the time of this analysis, 397 participants had provided a fasting blood sample in adulthood (18–39 years) for the measurement of type 2 diabetes risk markers. Additionally, participants fulfilled the eligibility criteria of being singletons, born at term (37 to <43 gestation weeks) with normal birthweight. To estimate habitual intake of dietary sugars during adolescence (females: 9–15 years, males: 10–16 years), participants additionally had to have provided at least two 3-day weighed dietary records in the period of adolescence (with >50% plausible records) (32) (*n* = 277) or at least two complete 24 h urine samples in adolescent years (*n* = 246) for the measurement of excreted fructose and sucrose, validated biomarkers of sugar intake (29, 33). The plausibility of dietary records was estimated by calculating the ratio between reported total energy intake and estimated basal metabolic rate (estimated according to age- and sex-specific equations of Schofield) (34). To identify energy underreporting, pediatric cutoffs from Sichert-Hellert et al. were used (32). Underreporters were not excluded from the analyses, as this procedure only identifies underreported energy intake, but no selective underreporting of food groups or sugar intake. Instead a sensitivity analysis excluding energy underreporters was performed. Anthropometric measurements from adolescence and adulthood as well as information on relevant covariates and outcome variables were required, resulting in analysis populations of 254 participants for the dietary intake sample and 221 participants for the HOMA-%S biomarker sample (see Tables 1, 2) (with *n* = 220 providing both dietary and biomarker data). The inflammatory score sample population differed slightly (*n* = 253 in dietary sample, and *n* = 219 in the biomarker sample). Participants with fasting glucose concentrations above the threshold (>2.5 mmol/L) for calculating HOMA2-%S were included in the analysis (*n* = 254).

**Table 1:** Baseline characteristics of DONALD participants in adolescence (males: 10–16 years, females: 9–15 years): anthropometry, dietary and urinary data as well as early life and socioeconomic factors.

	**Dietary sample**			**Urinary sample**		
	***n***	**M (*n* = 124)**	**F (*n* = 130)**	***n***	**M (*n* = 109)**	**F (*n* = 112)**
**Age** (years)	254	13.0 (13.0, 13.1)	12.0 (11.9, 12.0)	221	13.0 (13.0, 13.0)	12.0 (12.0, 12.0)
**Anthropometric data**						
BMI-SD score	254	−0.18 ± 0.77	−0.23 ± 0.92	221	−0.16 ± 0.80	−0.22 ± 0.93
BMI (kg/m^2^)	254	18.8 (17.7, 20.2)	17.8 (16.5, 20.1)	221	19.1 (17.7, 20.3)	17.9 (16.5, 20.3)
Body fat (%)	254	14.8 (11.6, 18.6)	19.6 (16.8, 24.9)	221	15.2 (11.6, 18.8)	19.6 (16.9, 25.3)
Overweight (%)^a^	254	22.6	22.3	221	25.7	22.3
**Dietary data**						
Total energy (MJ/d)	254	9.0 (8.1, 10.2)	7.1 (6.6, 8.1)	221	9.0 (8.3, 10.2)	7.2 (6.6, 8.1)
Fat (%E)	254	35.3 ± 3.8	36.1 ± 3.5	221	34.9 ± 3.4	36.2 ± 3.5
Protein (%E)	254	13.2 ± 1.3	12.9 ± 1.7	221	13.2 ± 1.3	12.9 ± 1.7
Fiber (g/MJ)	254	2.4 (2.1, 2.7)	2.5 (2.2, 2.8)	221	2.4 (2.2, 2.8)	2.5 (2.1, 2.8)
Carbohydrate (%E)	254	51.3 ± 3.8	51.1 ± 4.3	221	51.5 ± 3.9	51.1 ± 4.4
Total sugar (%E)	254	26.8 ± 5.0	27.1 ± 5.0	221	27.0 ± 5.1	27.0 ± 4.9
Added sugar (%E)	254	14.3 ± 4.3	14.1 ± 4.7	221	14.2 ± 4.4	14.1 ± 4.7
Free sugar (%E)	254	18.2 ± 4.6	17.7 ± 5.0	221	18.4 ± 4.6	17.6 ± 5.0
Sucrose (%E)	254	14.4 ± 3.8	14.6 ± 3.9	221	14.4 ± 3.8	14.5 ± 3.8
Fructose (%E)	254	11.3 ± 2.6	11.4 ± 2.5	221	11.4 ± 2.6	11.3 ± 2.4
Glucose (%E)	254	11.5 ± 2.5	11.8 ± 2.7	221	11.4 ± 2.5	11.8 ± 2.7
**Sources of total sugar**						
Juice (%E)	254	4.0 ± 3.5	3.6 ± 2.9	221	4.3 ± 3.6	3.5 ± 2.9
SSB (%E)	254	4.5 ± 3.9	3.9 ± 3.7	221	4.4 ± 3.8	4.0 ± 3.5
Fruits and vegetables (%E)	254	3.2 ± 1.8	4.3 ± 1.9	221	3.2 ± 1.8	4.3 ± 1.9
Sweet breads/cakes (%E)	254	1.2 ± 0.8	1.4 ± 0.8	221	1.3 ± 0.8	1.4 ± 0.9
Sweets/sugar (%E)	254	6.2 ± 2.5	7.0 ± 2.7	221	6.0 ± 2.4	6.9 ± 2.6
Sweetened cereals (%E)	254	1.0 ± 0.9	0.7 ± 0.6	221	1.1 ± 1.1	0.7 ± 0.7
Dairy sugars (%E)	254	5.3 ± 2.5	4.8 ± 2.2	221	5.5 ± 1.5	4.8 ± 1.0
**Urinary data**						
Urinary fructose (mg/d)				221	22.3 (14.5, 32.3)	21.2 (13.4, 32.3)
Fructose+sucrose (mg/d)				221	52.7 (37.2, 79.0)	46.3 (34.4, 68.2)
Creatinine (mmol/L)				221	9.5 (6.7, 11.5)	7.6 (6.0, 10.0)
Urea (mmol/L)				221	323 (255, 416)	272 (216, 349)
Urine Volume (L/d)				221	0.9 (0.7, 1.3)	1.0 (0.7, 1.2)
**Early life/socioeconomic data**						
Birth weight (g)	254	3500 (3150, 3845)	3405 (3100, 3700)	221	3550 (3180, 3850)	3400 (3100, 3655)
Gestational age (week)	254	40 (39, 41)	39 (38, 41)	221	40 (39, 41)	40 (39, 41)
Gestational weight gain (kg)	254	12.0 (9.5, 14.5)	12.0 (9.0, 15.0)	221	12 (10, 15)	12 (10, 15)
Maternal age at birth (year)	254	30.7 (28.3, 33.7)	30.0 (27.8, 32.7)	221	30.8 (28.3, 33.6)	29.7 (27.7, 32.6)
Full breastfeeding >2 weeks (%)	254	74	73	221	75	76
Paternal education ≥12 y (%)	254	65	57	221	64	57
Any smokers in household (%)	254	27	37	221	28	37

*Values are means ± SD, medians (25th, 75th percentile) or relative frequencies. BMI, body mass index; %E = percentage of total energy intake; DONALD Dortmund Nutritional and Anthropometric Longitudinally Designed; Pubertal age: mean age at pubertal data collection (mean of multiple time points)*.

a *Defined according to age- and sex-specific cut points of the International Obesity Task Force (1, 35); Dietary fructose intake is defined to be free fructose plus 50% of sucrose. Dietary glucose intake is defined to be free glucose plus 50% of sucrose*.

**Table 2:** Follow-up data on DONALD participants in early adulthood (18–36 years): anthropometric and lifestyle, dietary and blood data.

	**Dietary sample**			**Urinary sample**		
	***n***	**M (*n* = 124)**	**F (*n* = 130)**	***n***	**M (*n* = 109)**	**F (*n* = 112)**
**Adult age** (years)	254	20.5 (18.1, 23.0)	21.3 (18.1, 24.2)	221	19.0 (18.1, 23.0)	21.3 (18.1, 24.2)
**Anthropometric data**						
BMI (kg/m^2^)	253	22.7 (21.1, 25.6)	21.9 (20.5, 24.1)	221	22.7 (21.0, 25.6)	21.9 (20.5, 24.1)
Body fat (%)	253	17.2 (13.4, 22.2)	30.4 (27.2, 33.3)	221	17.4 (13.3, 21.9)	30.5 (27.0, 33.2)
Current smoking (%)	235	36.8	32.7	202	32.3	28.4
Physical activity level^a^	252	1.2 (1.1, 1.4)	1.2 (1.1, 1.2)	220	1.2 (1.1, 1.4)	1.2 (1.1, 1.3)
Alcohol intake (g/d)	228	1.3 (0.01, 12.5)	0.2 (0.0, 2.9)	203	1.4 (0.1, 11.6)	0.3 (0.1, 3.0)
**Dietary data**						
Total energy (MJ/d)	229	10.6 (9.3, 12.5)	7.9 (6.6, 8.8)	203	10.5 (9.3, 12.4)	8.0 (6.7, 9.0)
Added sugar (%E)	229	13.3 ± 6.8	12.7 ± 7.4	203	13.4 ± 7.4	12.8 ± 7.2
Protein (%E)	229	14.3 ± 3.8	13.5 ± 2.6	203	14.5 ± 3.9	13.4 ± 2.2
Carbohydrates (%E)	229	48.6 ± 6.7	51.0 ± 6.4	203	48.8 ± 7.0	51.0 ± 6.1
Fat (%E)	229	36.0 ± 5.0	34.6 ± 4.7	203	36.3 ± 5.1	34.9 ± 5.9
Fiber (g/MJ)	229	2.4 (2.0, 2.9)	2.2 (1.9, 2.7)	203	2.2 (1.9, 2.7)	2.5 (2.2, 3.0)
**Blood data**						
Fasting blood glucose (mmol/L)	254	5.5 (5.1, 5.8)	5.2 (4.9, 5.4)	221	5.5 (5.1, 5.8)	5.2 (4.9, 5.4)
Insulin (pmol/L)	254	64.1 (52.7, 85.5)	71.4 (55.4, 88.2)	221	64.0 (52.1, 85.8)	72.9 (57.3, 89.4)
HOMA2-%S	254	81.7 (61.7, 100.4)	73.5 (960.5, 94.2)	221	81.8 (60.9, 100.6)	73.0 (60.5, 93.9)
hsCRP (mg/L)	250	0.5 (0.3, 1.1)	1.2 (0.6, 2.6)	217	0.5 (0.3, 1.3)	1.3 (0.6, 2.7)
IL-6 (pg/mL)	250	0.7 (0.5, 1.0)	0.7 (0.5, 1.0)	217	0.7 (0.5, 1.0)	0.7 (0.5, 1.0)
IL-18 (pg/mL)	250	252 (204, 308)	246 (209, 306)	217	249 (204, 303)	247 (207, 306)
Chemerin (ng/mL)	250	141 (123, 160)	165 (150, 184)	217	141 (123, 159)	165 (150, 183)
Leptin (ng/mL)	250	2.4 (1.2, 5.0)	11.6 (7.8, 18.0)	217	2.3 (1.1, 5.1)	11.7 (7.8, 18.2)
Adiponectin (μg/mL)	250	6.2 (4.5, 9.2)	8.7 (6.5, 12.5)	217	6.4 (4.7, 9.2)	8.7 (6.4, 12.9)
Inflammatory score	250	−0.13 (−0.37, 0.28)	−0.07 (−0.38, 0.37)	217	−0.15 (−0.37, 0.26)	−0.06 (−0.38, 0.38)

*Values are means ± SD, medians (25th, 75th percentile) or relative frequencies. BMI, body mass index; %E = percentage of total energy intake; DONALD Dortmund Nutritional and Anthropometric Longitudinally Designed; HOMA2-%S updated homeostasis model assessment of insulin sensitivity, hsCRP high-sensitivity C-reactive protein*.

a *Based on energy expenditure levels*.

### Dietary Assessment

Dietary intake data of the participants are collected annually by 3-day weighed dietary records under the professional direction of a dietician. All consumed foods as well as leftovers were weighed to the nearest gram or alternatively are recorded semi-quantitatively if weighing was not possible. The calculation of energy and nutrient intakes that are based on dietary records is carried out by using the in-house food database called LEBTAB, which is continuously updated (31). The composition of staple foods is based on the German food composition tables BLS 3.02. Energy and nutrient contents of commercial food products, i.e., processed foods and ready-to-eat-meals were estimated by recipe simulation using labeled ingredients and nutrient contents. In this analysis, we calculated the intake of added, free, and total sugar, as well as fructose (defined as simple fructose + one-half of sucrose), glucose and sucrose. Total sugar was defined as the sum of all mono- and disaccharides in foods. Added sugar was defined as sugars added to foods during processing or home preparation (including honey, molasses, fruit juice concentrate, brown sugar, corn sweetener, sucrose, lactose, glucose, high-fructose corn syrup, and malt syrup). Because free sugar was not included in LEBTAB, we expanded the definition from the World Health Organization (WHO) of free sugar as suggested by the Scientific Advisory Committee on Nutrition (SACN) (25, 36) who states that “food subject to blending, pulping, or macerating which breaks down the cellular structure should also be considered as containing free sugars.” Therefore, sugars from juices, juice spritzers and smoothies were also considered to be free sugars in our study. Further, SSB were defined as sweetened fruit juice drinks and nectars, soft drinks/sodas, sweetened teas and waters, instant beverages (except dairy drinks), and sweetened sports drinks. Juices were defined as fruits and vegetable juices, juice spritzers, and smoothies. The sugar/sweets food group was defined as sugars and other sweeteners (including syrups), sweet spreads, sweets (candies) and marshmallows, chocolate and bars, ice cream, jelly, desserts, sweet sauces, and sweet baking ingredients. Individual dietary sugar intakes were averaged over the three recorded days. Habitual intake was described by calculating an individual mean from all available records during adolescence (2–7 records per person, mean = 6).

### Anthropometric Measurements

Anthropometric measurements were taken by trained nurses according to standard procedures. Standing height was measured to the nearest 0.1 cm (digital stadiometer: Harpenden Ltd., Crymych, UK) and body weight to the nearest 0.1 kg (electronic scale: Seca 753E, Seca Weighing and Measuring Systems, Hamburg, Germany). From these measurements, BMI SD scores (sex- and age-specifically standardized according to German references) (37) and overweight during adolescence were defined and calculated according to the International Obesity Task Force (35). Waist circumference was measured at the midpoint between the lower rib and iliac crest to the nearest 0.1 cm. Average coefficients of variation were obtained from annual quality checks for biceps, triceps, subscapular, and supra-iliacal skinfolds.

### Collection and Analysis of 24 h Urine Samples

Participants are requested to collect 24 h urine annually according to standardized instructions. The participants were asked to void their bladders upon getting up in the morning and this micturition was completely discarded. This sets the start of the collection which ends with voiding the bladder in the next morning. All micturitions from the 24 h sampling period were collected in provided Extran-cleaned (Extran, MA03, Merck Darmstadt, Germany) preservative-free 1 L plastic containers and stored immediately at ≤-12°C. After transport to the study center the samples were stored at −22°C until thawed for analysis. Completeness of 24 h urine collections was determined by measuring creatinine excretions assessed photometrically by the kinetic Jaffé procedure on a creatinine analyzer (Beckman-2; Beckman Instruments) (38). Participants are asked to collect a 24 h urine on the last day of the 3-day dietary record, but this is not always the case and some persons do not provide 24 h urines during some of the years.

Urinary fructose and sucrose excretions were measured in the laboratory of the Department of Food and Nutritional Sciences at the University of Reading using LC-MS and quantified using stable-isotope labeled internal standards (^13^C_12_-sucrose and ^13^C_6_-fructose, Sigma Aldrich, Gillingham, UK). After shipping on dry ice, urine samples were stored at −80°C until analysis and thawed at 4°C. Samples were separated by HPLC and detected by tandem mass spectrometry using a Quattro Ultima tandem quadrupole mass spectrometer (Micromass, Manchester, UK). The concentration range was 0.1–500 μmol/L (Fructose: 0.02–90.1 mg/L; sucrose: 0.03–171.2 mg/L). To calculate daily excretions concentrations were converted to mg/d by using the molar mass of fructose or sucrose and multiplied with the 24 h urine volume (39).

### Collection of Blood Parameters

Venous blood samples were drawn after an overnight fast, centrifuged at 4°C and stored at −80°C. The following blood analytes were measured at the German Diabetes Center: plasma high-sensitivity C-reactive protein (hsCRP) using the Roche/Hitachi Cobas c311 analyzer (Roche diagnostics, Mannheim, Germany), plasma high-sensitivity interleukin (IL)-6 with the Human IL-6 Quantikine HS, plasma adiponectin with the Human Total Adiponectin/Acrp30 Quantikine ELISA and serum leptin with the Leptin Quantikine ELISA kits all from R&D Systems (Wiesbaden, Germany), serum IL-18 with the Human IL-18 ELISA kit from MBL (Nagoya, Japan), and plasma chemerin with the Human Chemerin ELISA kit from BioVendor (Brno, Czech Republic). Plasma concentrations of insulin were analyzed at the Laboratory for Translational Hormone Analytics of the University of Giessen using an immunoradiometric assay (IRMA, DRG Diagnostics, Marburg, Germany) and the updated HOMA2-%S, a measurement of insulin sensitivity. HOMA2-%S was calculated by using the HOMA2 calculator (40). It is a reciprocal of HOMA2-IR (insulin resistance) and is a function of glucose metabolism driven by the action of insulin.

To examine the association of dietary sugar on chronic low-grade inflammation in the DONALD Study, the pro-inflammatory markers CRP, IL-6, IL-18, chemerin, and leptin and the anti-inflammatory adipose tissue hormone adiponectin were considered. These biomarkers of subclinical inflammation were selected because they are the most commonly measured inflammation-related biomarkers in clinical and epidemiologic studies with established associations with cardiometabolic diseases (41–45).

A pro-inflammatory score, assumed to be more predictive of inflammation than single markers (43), was obtained as follows: (1) standardization of each inflammatory parameter (hsCRP, IL-6, IL-18, chemerin, leptin, adiponectin) by sex (mean = 0, *SD* = 1), (2) assignment of a minus sign to the anti-inflammatory parameter adiponectin to align its impact with the pro-inflammatory parameters, and (3) averaging all. This index has been used in previous publications (46, 47).

### Assessment of Further Covariates

Additional covariates were assessed either at the child's admission into the study or at follow-up visits. Characteristics of birth were retrieved from the “Mutterpass” (a German standardized pregnancy and birth document). Child's parents were interviewed in order to collect familial information, disease history, socioeconomic status and other anthropometrical and medical examinations. Smoking status, high paternal educational status (≥12 years of schooling), and physical activity of the participants was also assessed by questionnaires.

### Statistical Analysis

Characteristics of the study population are presented as mean ± SD or median (25th, 75th percentile) for continuous variables and as absolute (relative) frequencies for categorical variables (see Tables 1, 2).

To achieve normal distribution in outcome variables we used log_e_ or square root transformations. Before calculating the individual means from available records or urines during adolescence, dietary variables were energy-adjusted by the residual method and standardized by age group and sex to account for age- and sex-dependent intake differences. Urinary excretion variables were also standardized by age group and sex but were not energy-adjusted so as to keep the dietary and urinary analyses separate, thereby avoiding the mixing of potential errors from dietary record assessments with biomarker measurements, as they are differently biased.

Prospective associations between dietary sugar intake (total sugar, added sugar, free sugar, sucrose, fructose, glucose, total sugar from SSB, juice, and sweets/sugar) or sugar excretion (fructose excretion, sucrose excretion, sum of both) during adolescence and risk markers of type 2 diabetes or inflammation in early adulthood were analyzed by multivariable linear regression models, using the transformed variables. Formal interaction analyses indicated a trend in sex-interactions for insulin sensitivity and excreted fructose biomarker level (*P*_interaction_ = 0.06); therefore, sex-stratified analyses were performed for all outcomes on both the dietary and the biomarker level in order to allow comparability.

Initial regression models (model A) included the predictors sugar intake (total, free, added, sucrose, fructose, or glucose) or urinary biomarkers (fructose or sum of both) as well as age at time of blood draw. Adjusted models (model B) were constructed by individual examination of potential influencing covariates and hierarchical inclusion (16) of those which substantially modified the predictor–outcome associations (≥10%) or significantly predicted the outcome. Potential confounding covariates considered in the hierarchical approach were (1) *early life factors* [birth weight (g), gestational age (week), maternal age at birth (year), full breastfeeding ≥ 4 months (yes/no), and gestational weight gain (kg)], (2) *socioeconomic factors and parental health status* [smokers in the household (yes/ no), paternal school education ≥12 years (yes/no), parental overweight (BMI ≥25 kg/m^2^ yes/no) and parental history of diabetes (yes/no)], (3) *predictor-specific adolescent data* [BMI, BMI-SD score, percent body fat, age, energy- and fructose-adjusted flavonoid intake and glycemic index, and energy-adjusted fiber intake in models with the dietary predictors sugar intake]. For biomarker analyses, urinary variables [24 h-creatinine excretion (mmol/d), 24 h-urea excretion (mmol/d), urine volume (L/d), excreted hippuric acid (mmol/d)] were also considered. In conditional models (model C) we additionally included adult body fat (%) to examine whether observed associations were independent of adult body composition. To retain comparability of results, models were adjusted identically for closely related outcomes (parameters of insulin sensitivity (fasting insulin, HOMA2-%S) and separately for the pro-inflammatory score) and the building of the models was done for the primary exposures, i.e., dietary fructose or excreted fructose and then used for analyses of the secondary exposures, i.e., free sugar, total sugar, etc. Results from regression analyses are presented as adjusted least-square means (95% CI) by tertiles of the respective predictor with *p*-values from models with the predictors as continuous variables.

Our main analyses did not include nutritional factors that provide energy so as to avoid presenting estimates that partially reflect the substitution of specific sugars for other macronutrients. Additional models were run that explicitly assess the effect of a substitution of various dietary sugar fractions for non-sugar carbohydrates, i.e., total carbohydrates (g) minus all mono- and disaccharides (g). To simulate substitution effects, total energy and the energy-bearing nutrients to be held constant (fats, plant/animal protein and sugar-containing carbohydrates) were included in the models (48). All results from substitution analyses are presented in Supplementary Material for fully adjusted models.

As mentioned in the methods section, adolescents are susceptible to underreporting energy intake, therefore records were checked for energy underreporting. The number of records in which energy levels were underreported was 209 (12.6%). These were collected from 109 participants, and were excluded for sensitivity analyses; i.e., sensitivity analyses were based on 1,446 records from 277 participants.

Additional sensitivity analyses in subsamples of participants who had provided the following data were performed in dietary/urinary models: (a) levels of adult physical activity (low/medium/high; *n* = 252/218), (b) adult alcohol consumption (g/d; *n* = 229/203), (c) adult smoking (no, yes, earlier; *n* = 235/202).

The SAS statistical software package version 9.2 (SAS Institute Inc., Cary, NC) was used for all statistical analyses. To account for potential multiple testing, *p* < 0.01 were considered to indicate statistical significance, *p* < 0.05 were considered to indicate a trend.

## Results

Characteristics of the participants at baseline and at follow-up are presented in Tables 1, 2, respectively. The median follow-up times between the mean age during adolescence and adulthood were 9.0 years in the dietary sample and 8.6 years in the urinary sample. Participants were characterized by an above-average socioeconomic status as measured by the high percentage of participants' fathers with an education level >12 years. Tertiles of fructose, sucrose, glucose, total sugar, free sugar and added sugar intakes as well as the urinary sugars are shown in Tables 3–**5**. For results on sources of sugar (total sugars from SSB, juice and sweets/sugar; see Supplementary Table 4).

**Table 3:** Sex-stratified prospective associations of total dietary fructose, sucrose, and glucose intake during adolescence with markers of insulin sensitivity in early adulthood [*n* = 254: (124 males, 130 females)].

	**Tertiles of fructose intake**				**Tertiles of glucose intake**				**Tertiles of sucrose intake**			
**Females**	**Low (T1)**	**Moderate(T2)**	**High (T3)**	***P_***trend***_***	**Low (T1)**	**Moderate(T2)**	**High (T3)**	***P_***trend***_***	**Low (T1)**	**Moderate(T2)**	**High (T3)**	***P_***trend***_***
Dietary sugar(g/d)^a^	36 (32; 43)	47 (44; 53)	63 (56; 70)		39 (32; 43)	50 (43; 58)	65 (58; 70)		46 (39; 55)	60 (53; 69)	83 (70; 89)	
**Insulin (pmol/L)**												
Model A	79.3 (69.5; 89.2)	80.3 (70.1; 90.5)	72.6 (62.7; 82.5)	**0.03**	85.5 (75.9; 95.2)	78.0 (67.9; 88.1)	68.7 (59.0; 78.3)	**0.05**	82.8 (73.0; 92.5)	80.1 (70.3; 89.9)	68.8 (58.7; 78.8)	**0.03**
Model B	76.3 (66.7; 85.9)	79.9 (70.0; 89.8)	72.4 (62.8; 82.0)	0.11	81.5 (71.4; 91.6)	76.8 (66.6; 87.0)	70.8 (61.0; 80.6)	0.25	77.9 (67.5; 88.2)	79.0 (69.3; 88.7)	71.5 (61.4; 81.6)	0.20
Model C (conditional)	76.6 (66.9; 86.3)	79.2 (69.2; 89.2)	72.7 (63.0; 82.4)	0.17	80.9 (70.9; 90.9)	77.5 (67.4; 87.6)	70.7 (61.0; 80.4)	0.36	78.7 (68.5; 89.0)	79.0 (69.5; 88.6)	70.7 (60.6; 80.7)	0.20
**HOMA2-%S**												
Model A	71.8 (65.0; 79.5)	71.7 (64.6; 79.6)	79.1 (71.5; 87.6)	**0.04**	67.8 (61.4; 74.9)	73.7 (66.5; 81.8)	81.6 (73.9; 90.1)	0.06	68.8 (62.3; 76.0)	73.7 (66.7; 81.5)	80.9 (73.0; 89.7)	**0.03**
Model B	74.2 (67.3; 81.8)	72.5 (65.6; 80.2)	78.9 (71.5; 87.0)	0.13	68.8 (62.3; 76.0)	73.7 (66.7; 81.5)	81.0 (73.0; 89.7)	0.28	72.7 (65.4; 80.7)	74.6 (67.6; 82.2)	78.3 (70.7; 86.8)	0.25
Model C (conditional)	78.1 (69.1; 88.3)	76.2 (67.8; 85.6)	86.6 (77.1; 97.2)	0.20	71.5 (64.6; 79.1)	74.1 (66.9; 82.1)	79.6 (72.2; 87.8)	0.39	72.0 (64.9; 79.8)	74.5 (67.7; 82.1)	79.1 (71.4; 87.5)	0.24
**Males**	**Low (T1)**	**Moderate(T2)**	**High (T3)**	***P**_***trend***_*	**Low (T1)**	**Moderate(T2)**	**High (T3)**	***P**_***trend***_*	**Low (T1)**	**Moderate(T2)**	**High (T3)**	***P**_***trend***_*
Dietary sugar (g/d)^a^	47 (41; 50)	62 (52; 68)	79 (71; 89)		48 (38; 55)	61 (55; 72)	76 (67; 87)		58 (46; 69)	73 (62; 90)	99 (86; 114)	
**Insulin (pmol/L)**												
Model A	70.9 (60.9; 80.9)	78.3 (68.1; 88.4)	63.7 (53.8; 73.6)	0.95	65.4 (55.1; 76.9)	79.9 (68.8; 91.1)	71.8 (60.8; 82.8)	0.99	73.0 (62.5; 83.6)	72.8 (62.9; 82.6)	66.8 (56.6; 77.0)	0.41
Model B	71.0 (60.1; 81.9)	79.1 (68.7; 89.5)	64.2 (53.9; 74.5)	0.99	68.6 (57.5; 79.7)	72.5 (62.1; 83.0)	72.7 (62.5; 82.9)	0.87	73.8 (62.2; 85.5)	73.5 (63.4; 83.5)	67.3 (56.7; 77.9)	0.79
Model C (conditional)	71.6 (60.1; 82.0)	78.7 (68.3; 89.1)	64.7 (54.5; 75.0)	0.96	68.3 (57.2; 79.4)	73.3 (62.8; 83.9)	72.2 (61.9; 82.4)	0.90	73.4 (61.7; 85.1)	74.3 (64.2; 84.4)	66.7 (56.1; 77.3)	0.75
**HOMA2-%S**												
Model A	77.8 (69.4; 87.1)	76.9 (68.5; 86.3)	86.5 (77.3; 96.8)	0.90	79.9 (71.0; 90.0)	84.9 (75.8; 95.0)	76.6 (68.5; 85.6)	0.98	75.2 (66.8; 84.7)	80.9 (72.4; 90.4)	84.8 (75.6; 95.1)	0.40
Model B	78.1 (69.0; 88.3)	76.7 (68.2; 86.2)	86.0 (76.5; 96.6)	0.95	75.2 (66.8; 84.7)	80.9 (72.4; 90.4)	84.8 (75.6; 95.1)	0.89	75.0 (65.8; 85.4)	80.2 (71.7; 89.8)	84.7 (75.3; 95.4)	0.76
Model C (conditional)	74.4 (67.6; 82.0)	72.0 (65.1; 79.5)	79.1 (71.9; 87.2)	0.89	80.8 (71.4; 91.5)	83.4 (74.2; 93.8)	77.1 (68.8; 86.4)	0.92	75.4 (66.3; 85.9)	79.3 (70.8; 88.7)	85.5 (76.0; 96.2)	0.72

*Values are adjusted least-squares means (95% CIs) unless otherwise indicated. Linear trends (P_trend_) were obtained in sex-stratified linear regression models with the transformed and energy-adjusted predictors dietary fructose, sucrose, and glucose adolescent intakes as continuous variables. Model A adjusted for adult age at time of blood draw. Model B, with outcomes HOMA2-%S and fasting insulin, additionally adjusted for paternal education, birth weight, gestational weight gain, smoking in the household, parental overweight and pubertal percent body fat. Model C, the conditional model, additionally adjusted for adult percent body fat for all predictors and outcomes. Transformations of variables for analysis: log_e_ for HOMA2-%S, fasting insulin, dietary sucrose and glucose; square root for dietary fructose. HOMA2-%S: updated homeostasis model assessment of insulin sensitivity*.

a *Values are unadjusted medians (25th, 75th percentile). Fructose intake is defined to be free fructose plus 50% of sucrose. Glucose intake is defined to be free glucose plus 50% of sucrose. Bold values indicate significant findings (p < 0.01) or trends (p < 0.05)*.

### Adolescent Sugar Intake and Adult Insulin and Insulin Sensitivity

Intakes of dietary fructose, glucose or sucrose in adolescence were not independently associated with adult HOMA2-S% or insulin levels (all p > 0.01, Table 3). Similarly, there were no independent associations between total, free, or added sugar as well as total sugar intakes from SSB, juice, and sweets/sugar in adolescence and adult HOMA2-S% or insulin levels (all p > 0.01, Table 4).

**Table 4:** Sex-stratified prospective associations of total dietary sugar, added sugar, and free sugar intake during adolescence with markers of insulin sensitivity in early adulthood [n = 254: (124 males, 130 females)].

	**Tertiles of total sugar intake**				**Tertiles of added sugar intake**				**Tertiles of free sugar intake**			
**Females**	**Low (T1)**	**Moderate(T2)**	**High (T3)**	**P_**trend**_**	**Low (T1)**	**Moderate(T2)**	**High (T3)**	**P_**trend**_**	**Low (T1)**	**Moderate(T2)**	**High (T3)**	**P_**trend**_**
Dietary sugar(g/d)^a^	88 (79; 105)	113 (103; 129)	143 (128; 160)		39 (33; 47)	61 (50; 73)	78 (70; 95)		52 (43; 60)	76 (70; 86)	103 (98; 121)	
**Insulin (pmol/L)**												
Model A	81.3 (71.5; 91.0)	80.2 (70.1; 90.3)	70.7 (60.9; 80.6)	**0.05**	84.0 (74.2; 93.7)	76.9 (66.9; 86.8)	71.0 (61.0; 81.0)	0.24	81.1 (71.3; 90.9)	78.8 (68.6; 88.9)	72.3 (62.4; 82.2)	0.28
Model B	77.7 (67.6; 87.7)	78.8 (68.7; 88.9)	72.2 (62.4; 81.9)	0.23	79.2 (68.8; 89.6)	75.8 (66.0; 85.7)	73.5 (63.3; 83.6)	0.79	76.7 (66.7; 86.6)	78.7 (68.6; 88.7)	73.2 (63.4; 83.0)	0.51
Model C (conditional)	76.6 (66.6; 86.6)	80.5 (70.3; 90.6)	71.7 (62.0; 81.4)	0.28	79.6 (69.3; 89.9)	76.3 (66.6; 86.1)	72.6 (62.5; 82.7)	0.66	76.8 (67.0; 86.7)	79.1 (69.1; 89.0)	72.7 (63.0; 82.4)	0.54
**HOMA2-%S**												
Model A	71.1 (64.4; 78.6)	71.3 (64.3; 79.1)	80.5 (72.7; 89.0)	0.06	68.5 (62.0; 75.7)	75.7 (63.3; 83.9)	79.0 (71.3; 87.5)	0.28	70.8 (64.1; 78.3)	72.5 (65.4; 80.5)	79.4 (71.7; 87.9)	0.32
Model B	74.2 (67.0; 82.1)	72.3 (65.3; 80.1)	78.9 (71.5; 87.1)	0.27	72.5 (65.2; 80.5)	76.4 (69.2; 84.4)	76.6 (69.1; 84.9)	0.87	74.6 (67.5; 82.5)	72.3 (65.4; 80.0)	78.5 (71.1; 86.6)	0.57
Model C (conditional)	75.0 (67.9; 83.0)	71.0 (64.1; 78.6)	79.4 (72.0; 87.5)	0.33	72.1 (65.0; 80.1)	76.0 (68.9; 83.9)	77.3 (69.9; 85.6)	0.73	74.5 (67.5; 82.2)	72.0 (65.2; 79.6)	78.9 (71.6; 87.0)	0.61
**Males**	**Low (T1)**	**Moderate(T2)**	**High (T3)**	**P**_**trend**_	**Low (T1)**	**Moderate(T2)**	**High (T3)**	**P**_**trend**_	**Low (T1)**	**Moderate(T2)**	**High (T3)**	**P**_**trend**_
Dietary sugar (g/d)^a^	110 (95, 128)	148 (135, 172)	173 (150, 200)		51 (42, 68)	73 (67, 89)	102 (86, 125)		66 (55, 77)	92 (81, 111)	129 (110, 141)	
**Insulin (pmol/L)**												
Model A	70.3 (60.0; 80.6)	74.5 (64.4; 84.5)	67.7 (57.5; 77.9)	0.91	65.9 (55.4; 76.4)	76.9 (67.2; 86.6)	68.9 (58.9; 79.0)	0.92	68.4 (57.8; 78.9)	76.2 (66.4; 86.1)	67.6 (57.6; 77.6)	0.28
Model B	70.5 (59.4; 81.5)	75.6 (65.3; 86.0)	67.9 (57.4; 78.4)	0.86	66.6 (55.6; 77.6)	76.6 (66.6; 86.6)	70.1 (59.7; 80.5)	0.72	69.0 (57.5; 80.4)	76.4 (66.3; 86.6)	68.6 (58.3; 78.9)	0.78
Model C (conditional)	70.5 (59.5; 81.5)	76.0 (65.7; 86.3)	67.5 (57.0; 78.0)	0.87	66.6 (55.6; 77.6)	76.8 (66.8; 86.8)	69.8 (59.4; 80.2)	0.79	68.5 (57.0; 79.9)	77.2 (67.0; 87.4)	68.1 (57.9; 78.4)	0.89
**HOMA2-%S**												
Model A	78.8 (70.2; 88.5)	79.5 (71.0; 89.1)	82.8 (73.8; 92.9)	0.85	84.0 (74.3; 94.5)	76.5 (68.5; 85.4)	81.4 (72.6; 91.2)	0.97	80.4 (71.4; 90.6)	77.8 (69.6; 87.0)	83.0 (74.1; 93.0)	0.93
Model B	79.2 (70.0; 89.7)	78.6 (70.0; 88.3)	83.0 (73.7; 93.3)	0.93	83.9 (74.1; 95.0)	77.2 (68.8; 86.4)	80.4 (71.5; 90.4)	0.69	80.4 (70.7; 91.5)	78.1 (69.7; 87.6)	82.1 (73.1; 82.2)	0.79
Model C (conditional)	79.2 (70.0; 89.7)	78.2 (69.6; 87.8)	83.5 (74.2; 93.9)	0.95	83.9 (74.2; 95.0)	77.0 (68.8; 86.1)	80.8 (71.9; 90.8)	0.77	81.0 (71.3; 92.1)	77.2 (68.9; 86.6)	82.7 (73.7; 92.8)	0.89

*Values are adjusted least-squares means (95% CIs) unless otherwise indicated. Linear trends (P_trend_) were obtained in sex-stratified linear regression models with the transformed and energy-adjusted predictors dietary fructose, sucrose, and glucose adolescent intakes as continuous variables. Model A adjusted for adult age at time of blood draw. Model B, with both outcomes HOMA2-%S and fasting insulin additionally adjusted for paternal education, birth weight, gestational weight gain, smoking in the household, parental overweight and pubertal percent body fat. Model C, the conditional model, additionally adjusted for adult percent body fat for all predictors and outcomes. Transformations of variables for analysis: log_e_ for HOMA2-%S, fasting insulin, total sugar intake; square root for added sugar and free sugar intakes. HOMA2-%S: updated homeostasis model assessment of insulin sensitivity*.

a *Values are unadjusted medians (25th, 75th percentile). Bold values indicate significant findings (p < 0.01) or trends (p < 0.05)*.

**Figure 1:**
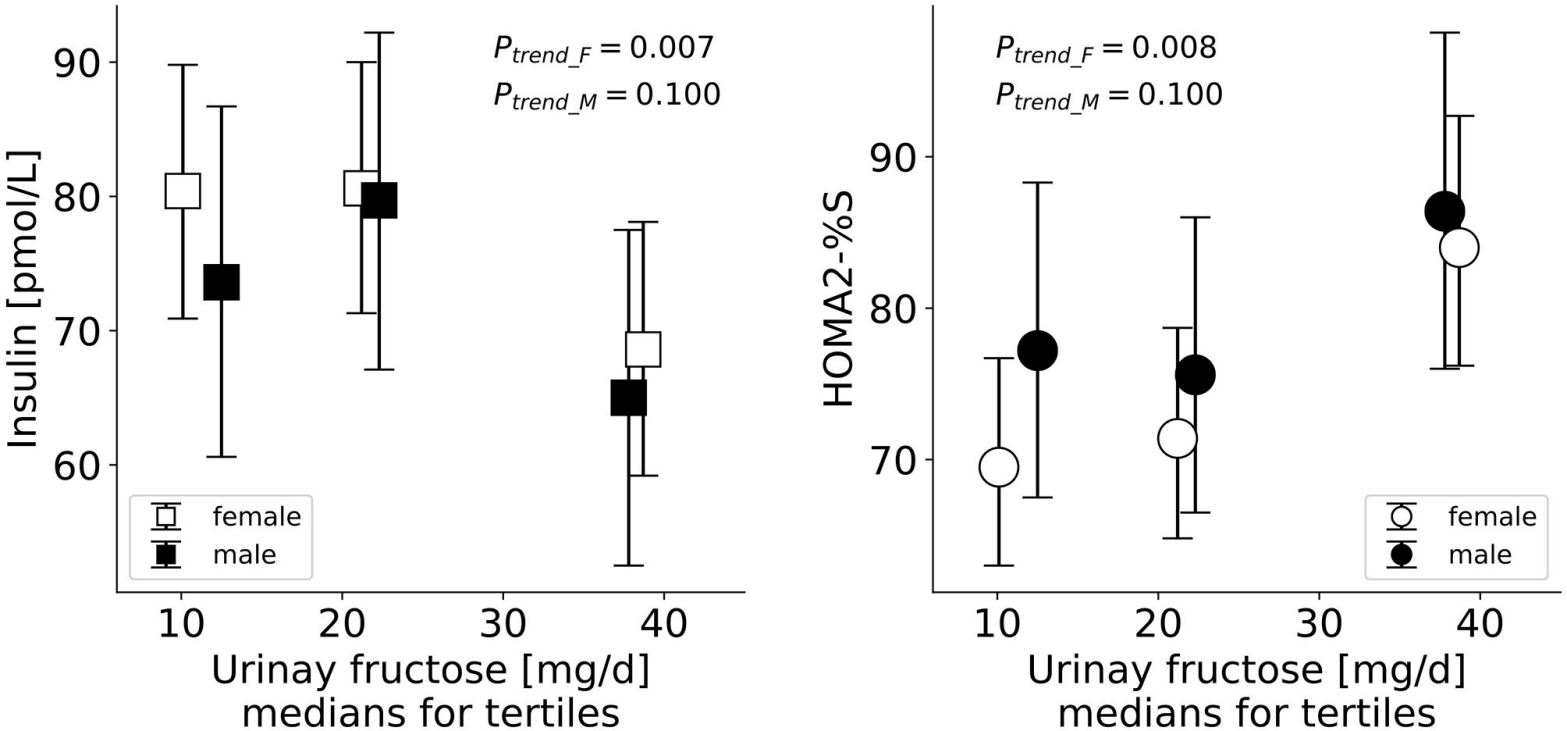
Serum levels of fasting insulin and insulin sensitivity (HOMA2-%S) in early adulthood by tertiles of excreted urinary fructose among females and males in adolescence. Data are generic means and 95% CI adjusted for age at time of blood draw, paternal education, pubertal percent body fat, gestational weight gain and adult percent body fat.

On the biomarker level, a higher adolescent excretion of urinary fructose was associated with lower fasting insulin and higher adult insulin sensitivity among females (*p* = 0.007 and *p* = 0.008, respectively, Table 5, model C; Figure 1). Among males, sugar excretion levels were not associated with adult insulin sensitivity markers.

**Table 5:** Sex-stratified prospective associations of urinary fructose, urinary sucrose, and the sum of urinary fructose and sucrose excretion during adolescence with markers of insulin sensitivity in early adulthood [(*n* = 221: (109 males, 112 females))].

	**Tertiles of urinary fructose**				**Tertiles of urinary fructose** **+** **sucrose**			
**Females**	**Low (T1)**	**Moderate (T2)**	**High (T3)**	***P_***trend***_***	**Low (T1)**	**Moderate (T2)**	**High (T3)**	***P_***trend***_***
Urinary sugar (mg/d)^a^	10.1 (7.9, 13.3)	21.2 (19.0, 24.5)	38.7 (32.3, 54.8)		27.0 (21.7, 34.3)	46.1 (41.0, 52.7)	79.4 (67.4, 110.9)	
**Insulin (pmol/L)**								
Model A	80.6 (70.7; 90.4)	82.2 (72.4; 91.9)	69.4 (59.5; 79.2)	**0.013**	78.2 (68.2; 88.2)	78.0 (67.9; 88.2)	76.1 (66.3; 85.9)	0.29
Model B	79.7 (70.0; 89.4)	81.9 (72.3; 91.4)	67.8 (58.1; 77.5)	**0.011**	76.9 (67.0; 86.9)	77.0 (67.1; 86.9)	75.7 (66.1; 85.3)	0.24
Model C (conditional)	80.4 (70.9; 89.8)	80.6 (71.3; 90.0)	68.6 (59.2; 78.1)	**0.007**	76.2 (66.6; 85.9)	79.0 (69.3; 88.8)	74.8 (65.5; 84.2)	0.18
**HOMA2-%S**								
Model A	69.3 (62.5; 76.9)	70.5 (63.6; 78.1)	83.0 (74.8; 92.1)	**0.015**	71.7 (64.4; 79.7)	73.9 (66.3; 82.4)	76.3 (68.8; 84.7)	0.31
Model B	70.0 (63.3; 77.4)	70.6 (64.0; 78.0)	84.7 (76.6; 93.6)	**0.013**	72.5 (65.4; 80.5)	74.9 (67.5; 83.1)	76.7 (69.3; 84.8)	0.25
Model C (conditional)	69.5 (63.0; 76.7)	71.4 (64.8; 78.7)	84.0 (76.2; 92.7)	**0.008**	73.0 (66.0; 80.9)	73.5 (66.3; 81.5)	77.3 (70.0; 85.3)	0.19
**Males**	**Low (T1)**	**Moderate(T2)**	**High (T3)**	***P***_***trend***_	**Low (T1)**	**Moderate(T2)**	**High (T3)**	***P***_***trend***_
Urinary sugar (mg/d^a^	12.5 (9.9, 14.2)	22.3 (18.3, 23.2)	37.8 (32.5, 51.7)		31.6 (24.8, 37.1)	52.0 (44.7, 56.0)	89.7 (75.7, 117.8)	
**Insulin (pmol/L)**								
Model A	72.5 (59.8; 85.3)	79.9 (67.6; 92.1)	65.2 (52.7; 77.8)	0.20	76.2 (63.6; 88.9)	74.6 (62.1; 87.1)	67.2 (54.4; 79.9)	0.53
Model B	72.2 (59.4; 85.0)	80.7 (68.3; 93.1)	65.6 (53.2; 78.1)	0.23	75.2 (62.3; 88.0)	76.0 (63.4; 88.6)	67.3 (54.7; 80.0)	0.74
Model C (conditional)	73.6 (60.6; 86.7)	79.7 (67.1; 92.2)	65.0 (52.5; 77.5)	0.10	76.6 (63.7; 89.5)	77.1 (63.6; 88.7)	65.5 (52.6; 78.3)	0.18
**HOMA2-%S**								
Model A	79.0 (69.1; 90.2)	74.6 (65.7; 84.8)	85.6 (75.1; 97.6)	0.20	76.4 (66.9; 87.1)	78.7 (69.1; 89.6)	83.7 (73.3; 95.6)	0.50
Model B	79.4 (69.5; 90.8)	74.1 (65.1; 84.4)	85.4 (75.0; 97.2)	0.23	77.4 (67.7; 88.4)	77.6 (68.1; 88.5)	83.7 (67.7; 88.4)	0.71
Model C (conditional)	77.2 (67.5; 88.3)	75.6 (66.5; 86.0)	86.4 (76.0; 98.2)	0.10	75.5 (66.1; 86.1)	77.5 (68.2; 88.1)	86.4 (75.8; 98.5)	0.29

*Values are adjusted least-squares means (95% CIs) unless otherwise indicated. Linear trends (P_trend_) were obtained in sex-stratified linear regression models with the predictors urinary fructose, urinary sucrose, and sum of urinary fructose and sucrose as continuous variables. Model A adjusted for adult age at time of blood draw. Model B, with outcomes HOMA2-%S and fasting insulin, additionally adjusted for paternal education, pubertal percent body fat and gestational weight gain. The conditional Model C additionally adjusted for adult percent body fat for all predictors and outcomes. Transformations of variables for analysis: log_e_ for HOMA2-%S, fasting insulin; square root for excreted urinary fructose; log_e_(log_e_) for sum of excreted fructose and sucrose. HOMA2-%S: updated homeostasis model assessment of insulin sensitivity*.

a *Values are unadjusted medians (25th, 75th percentile). Bold values indicate significant findings (p < 0.01) or trends (p < 0.05)*.

### Adolescent Sugar Intake and Adult Systemic Inflammation

Intakes of glucose, fructose, sucrose, total sugar, free sugar or added sugar as well as total sugar intakes from SSB, juice and sweets/sugar were not independently associated with the pro-inflammatory score in adulthood (all *p* > 0.01; see Supplementary Tables 1, 2, 4). Similarly, sugar excretion levels during adolescence were not associated with the pro-inflammatory score in adulthood (all *p* > 0.01, Supplementary Table 3).

### Sensitivity Analyses

All sensitivity analyses yielded similar results as the main investigation, i.e., did not significantly change any observed associations. The results from the substitution analyses indicate that the replacement of each sugar type for non-sugar carbohydrates did not result in any significant associations for the outcomes of pro-inflammatory score (see Supplementary Table 5), fasting insulin and insulin sensitivity (see  Supplementary Table 6).

## Discussion

In the present longitudinal study, a unique database compiled from self-reported sugar intake data and urinary fructose and sucrose excretion as dietary sugar intake biomarkers was used to investigate the role of dietary sugars in adolescence for adult risk markers of T2D. The main finding suggests that dietary sugar was not consistently related to adult T2D risk factors. The only exception was the urinary fructose biomarker, which was beneficially associated with HOMA2-S% and fasting insulin levels among females only. No other associations were found between the various dietary/urinary sugars and insulin sensitivity or chronic inflammation.

Other reported observational evidence was consistent with our prospective association between fructose intake and improved HOMA2-S% and insulin levels (49, 50), and further sources reporting on large cohorts found no association between fructose-containing sugars and incident T2D (51–53) contrary to the popular opinion that sugar intake increases risk for T2D. A meta-analysis of 15 prospective cohort studies reported no association of total sugar and fructose intake with T2D, and a higher sucrose consumption was associated with a decreased risk in T2D (54). The main predictors in these studies reported findings that emerged when investigating chemical sugar types, as was similarly done in our study. The observational studies referenced here similarly adjusted for anthropometric measures and energy intake as was done in our study but did not measure sugar intake by means of urinary biomarkers. When consumed in high amounts, dietary fructose has been associated in cohort studies with increased risk of T2D (55, 56). Inconsistent findings related to sugar intake and diabetes risk may result from varying levels of sugar intake and the possibility that different sugars elicit different metabolic effects (57). Our results pertaining to biomarkers of inflammation indicated no relationship with sugar intake. Only when analyzing sugar as a source of SSB was it associated with an increased pro-inflammatory score among females (*P*_trend_ < 0.05). This is consistent with observational evidence that consistently links SSB intake with increased chronic inflammation (more specifically CRP) (1, 20–22).

There is an array of categories and uses by which dietary sugar is defined and tested for in nutritional research. Broken down on a chemical level, the monosaccharides fructose and glucose and the disaccharide sucrose are assumed to have unique metabolic effects on outcomes of health. Other sugar categories of total, added, or free sugars may each be of physiological relevance, i.e., causing varying effects on absorption, satiety, caloric compensation, or insulin response. Since dietary assessment methods are prone to measurement errors (26) and sugars are among the nutrients that are frequently underreported (27, 28), objective dietary biomarkers of 24 h urinary sucrose and urinary fructose have been introduced (29, 30). The inconsistencies often found in epidemiological studies that investigate links between sugars and chronic disease may in part be due to the ambiguity of not only the definition and type of sugar but the sugar source as well (9–11). When the main sources of dietary fructose are fruits and vegetables in their whole form and not as juice, prospective studies have shown inverse associations with the risk of incident diabetes (58, 59). This may be related to factors specifically associated with fruit and vegetable intake, such as particular micronutrients or dietary patterns that are related to a lower risk of diabetes. Although fruit/vegetable juices contain bioactive compounds such as vitamins and phytochemicals, they are stripped of the fiber once had in their whole food form and have sugar and energy contents similar to SSB (60). Additionally, liquid sources of sugar affect satiety differently than solid sources (61). A distinction is made between different types of fruit juices; sugar-sweetened fruit juice has been reported to increase the risk of developing T2D in some prospective studies (55, 62), while in others 100% fruit juice showed no association (63–65) as confirmed by a meta-analysis (66). Sugar-sweetened fruit juice was defined as an SSB in our study, and our juice variable came from fruit and vegetable sources; no associations were observed for fruit juice intake in our study.

Our finding relating to the inverse association of urinary fructose on insulin levels is in line with evidence from short-term trials that reported decreases in circulating insulin in subjects consuming fructose-sweetened beverages compared to glucose-sweetened beverages (13, 14). Fructose consumption causes smaller excursions in insulin due to its inability to stimulate the secretion of insulin from pancreatic beta cells. This was also confirmed by a meta-analysis of randomized trials wherein iso-energetic replacements of glucose and sucrose with fructose resulted in decreased insulin levels (56). On the other hand, our finding that indicates a beneficial association of fructose intake with insulin sensitivity was not confirmed by many intervention studies in which high proportions of fructose are consumed. These fructose over-consumption trials almost consistently report that higher intakes of fructose lead to decreases in insulin sensitivity (67–70). Many of the studies outlining the biological pathways of fructose administer high levels of pure fructose and the observed outcomes are not applicable to the amount of fructose typically consumed by humans, particularly considering that fructose is most often co-ingested with glucose *via* sucrose or HFCS in ratios similar to sucrose. The human diet rarely encounters fructose as a single nutrient. When looking at the effects of small doses of fructose, a meta-analysis reported that small fructose intakes in iso-energetic exchange improves HbA1c and fasting blood glucose but had no effect on insulin resistance (71). When assessing the effect of dietary fructose, a distinction needs to be made between trials that administer high vs. low doses. Of note, our DONALD population consumed relatively low amounts of fructose. Thus, the comparisons made between our findings and those above are not helpful in explaining our results, also because we investigated longer-term relevance which is different from a short- or medium-term response to fructose consumption (evidence from available randomized controlled trials) unless a metabolic adaptation occurs during adolescence. Considering adult dietary sugar intake in our population, it was unrelated to both the outcomes and the predictors and thus did not change the findings.

In considering why it was only among females that the beneficial association of fructose was observed, other DONALD studies also reported that females were more influenced by dietary changes than men (72, 73). It has been reported that women show more dramatic changes than men in hormones and body composition due to reproductive factors, which may cause them to react more sensitively to changes in dietary influences. Differences between men and women are biology-linked and caused by differences in sex chromosomes, hormones, and gene expression of sex-specific autosomes, which can each have effects on organ systems (74). Especially during adolescence when the fuel economy shifts away from fatty acid composition and ketogenesis toward carbohydrate oxidation, there is reduced metabolic flexibility making puberty a vulnerable period for changes in body composition (75). Women generally have lowered insulin sensitivity (75–77) (as was also observed in this present study) or increased impaired glucose tolerance than do males (74), which may increase their susceptibility or sensitivity to dietary influences.

Sugars are often among the nutrients that are frequently misreported and perceived negatively because they are a source of empty calories and are a common ingredient in unhealthy foods (27, 28). A possible explanation in the present analysis for the contrasting regression results between dietary fructose and urinary fructose is selective underreporting of sugar-rich foods, e.g., sugar sweetened beverages or sweets. There is to date no reliable method to identify selective sugar underreporting. Our sensitivity analyses excluding underreporters of energy intake, i.e., dietary records that had implausible energy intake values, yielded similar results. The use of urinary biomarkers to estimate dietary sugar intake may produce more reliable results as they are less subject to measurement and misreporting errors. The inconsistency in the reported findings of observational studies that investigate relations between sugar and disease outcomes may be due to the ambiguity of the employed dietary assessment methods. This being said, weighed dietary records as used by the DONALD study have been considered to be the most accurate dietary assessment tool for larger study populations, and measurement errors using these records are smaller than for other methods of assessment (78, 79). Evidence based on self-reported intake, however, may be considered lower-grade when compared to objective dietary biomarkers, especially due to selective underreporting of unhealthy foods (80). Neither fructose nor sucrose is endogenously synthesized, therefore urinary excretion has to be of dietary origin. A small amount of sucrose escapes from enzymatic hydrolysis in the small intestine and enters into blood stream before becoming excreted. For ingested fructose, a small proportion derived from free fructose and from hydrolysis of sucrose escapes hepatic fructose metabolism and is likewise excreted through the urine. In the existing literature it is still debated which sugars (extrinsic, intrinsic, total, added, free, etc.) are really captured by urinary sucrose and fructose excretion (29, 30, 81, 82). In a previous DONALD publication, it was found that dietary total sugar was more strongly associated with excreted fructose than dietary added sugar (83). While the relationship between intake and excretion is more complex for 24 h urinary sugars than for recovery biomarkers, they have been shown to reflect intake as so-called predictive biomarkers. Following extensive validation data, Tasevska et al. (84) have shown that it is possible to estimate actual intake from these markers when one considers age and sex. Both dietary and biomarker methods of assessment are analyzed and compared in this study; they have different sources of error and do not necessarily cover the exact same days of assessment (rather the same overall time period).

The main strength of the present study was its longitudinal design, including the long follow-up, which allowed the investigation of the long-term associations between dietary sugar intake in adolescence outcomes in young adulthood. Unlike many other observational studies of this nature, it was a strength that our study allowed comparisons of associations on the dietary as well as the urinary level. The urinary biomarkers are less subject to confounding by other nutrients or underreporting. In addition, our continuously updated in-house nutrient database LEBTAB allowed the consideration of fructose, glucose and sucrose as well as different types of fructose-containing sugars (total sugar, free sugar, added sugar). Our study was able to consider brand-specific sugar content in commercial products as well as sugars or sweetening agents such as syrups and honey which are used for food preparation at home. Furthermore, the urine analyses were carried out in established laboratories by scientists with years of experience in the measurement of sugar excretion in 24 h-urine samples.

Our study was limited by the availability of only one blood sample in young adulthood. It could be argued that the follow-up time was rather short considering the younger age of the cohort, and therefore endpoints of incidence could not be assessed. Since T2D rates occur ever increasingly in younger populations we justified the decision to measure risk factors for T2D already in early adulthood. A further limitation in the methods used was the handling of our urine samples, which in contrast to previous studies (29, 30) were frozen without preservatives for a long period of time (the earliest 24 h urine was collected in 1985), which may have caused sucrose hydrolysis. Such a possible hydrolysis of sucrose would, however, query the successful application of urinary sucrose as a biomarker in large epidemiological studies in which urine samples are mostly stored without preservatives. Luceri et al. (58) were the first to examine urinary biomarkers for sugar intake referring only to the instability of sucrose in urine samples stored at room temperature. Since our samples were stored at < −12°C during the collection period at home as well as at −22°C in the study institute, our samples remained frozen until use. The generalizability of our results was limited due to the relatively high SES of the DONALD study population and high SES is known to correlate with lower dietary sugar intake (85). Nevertheless, our sugar intake data were similar to sugar intake in representative German nutrition survey (86, 87) as well as our sugar excretion data, which were similar to sugar excretion in other study populations (33, 88, 89).

In conclusion, these observational findings did not confirm that dietary sugar consumption in adolescence is related to insulin sensitivity in adulthood. The one potential exception to this was dietary fructose (as measured by a urinary fructose biomarker), which had a beneficial association with HOMA2-S% and fasting insulin levels among females in the context of a moderate fructose consumption pattern. No other associations were found between the various dietary/urinary sugars and insulin sensitivity or systemic inflammation.

## Data Availability Statement

The raw data supporting the conclusions of this article will be made available by the authors, without undue reservation.

## Ethics Statement

The studies involving human participants were reviewed and approved by The Ethics Committee of the University of Bonn, Germany. Written informed consent to participate in this study was provided by the participants' legal guardian/next of kin.

## Informed Consent

All assessments in the DONALD study were performed with parental and later with participants written informed consent.

## Author Contributions

AB, UA, and TR conceived the research project. CH and MR supervised laboratory measurements of blood analytes. GK carried out the sugar analyses of the urine samples. SW measured the insulin and glucose levels. KP prepared parts of the data set. KDC conducted the statistical analysis and wrote the manuscript. AB supervised the project and had primary responsibility for the final content. All authors made substantial contributions, critically read and revised the manuscript as well as approved the final version.

## Conflict of Interest

AB is a member of the International Carbohydrate Quality Consortium (ICQC) and a member of the Carbohydrate Task Force, ILSI Europe. GK received a funding from Mars, Inc. for unrelated research on flavan-3-ols. The remaining authors declare that the research was conducted in the absence of any commercial or financial relationships that could be construed as a potential conflict of interest.
