# Supplementary material for: The Prospective Association of Dietary Sugar Intake in Adolescence With Risk Markers of Type 2 Diabetes in Young Adulthood
Source: Front Nutr. 2021 Jan 18;7:615684. doi: 10.3389/fnut.2020.615684 (PMC7848860; doi:10.3389/fnut.2020.615684)
Supplement: Supplementary file 1 [file Data_Sheet_1.docx]

Supplementary Material

|  | ***Tertiles of Fructose Intake*** | | | | ***Tertiles of Glucose Intake*** | | | | ***Tertiles of Sucrose Intake*** | | | |
| --- | --- | --- | --- | --- | --- | --- | --- | --- | --- | --- | --- | --- |
| *Females* | **Low**  **(T1)** | **Moderate**  **(T2)** | **High**  **(T3)** | ***P_trend_*** | **Low**  **(T1)** | **Moderate**  **(T2)** | **High**  **(T3)** | ***P_trend_*** | **Low**  **(T1)** | **Moderate**  **(T2)** | **High**  **(T3)** | ***P_trend_*** |
| Dietary sugar (g/d)^a^ | 36  (32; 43) | 47  (44; 53) | 63  (56; 70) |  | 39  (32; 43) | 50  (43; 58) | 65  (58; 70) |  | 46  (39; 55) | 60  (53; 69) | 83  (70; 89) |  |
| **Inflammatory score** | | | | | | | | | | | | |
| Model A | 1.09  (0.92; 1.31) | 1.00  (0.83; 1.20) | 0.96  (0.80; 1.14) | 0.29 | 1.16  (0.97; 1.39) | 0.87  (0.73; 1.04) | 1.03  (0.87; 1.22) | 0.51 | 1.02  (0.85; 1.22) | 0.93  (0.78; 1.11) | 1.10  (0.92; 1.32) | 0.54 |
| Model B | 1.07  (0.90; 1.28) | 1.00  (0.84; 1.21) | 0.97  (0.81; 1.15) | 0.40 | 1.14  (0.94; 1.38) | 0.88  (0.74; 1.05) | 1.04  (0.88; 1.25) | 0.29 | 0.98  (0.81; 1.19) | 0.94  (0.79; 1.23) | 1.12  (0.93; 1.35) | 0.48 |
| Model C  (conditional) | 1.04  (0.89; 1.21) | 1.07  (0.91; 1.25) | 0.96  (0.82; 1.11) | 0.72 | 1.09  (0.93; 1.29) | 0.86  (0.76; 1.03) | 1.08  (0.93; 1.26) | 0.62 | 1.02  (0.86; 1.20) | 0.93  (0.80; 1.08) | 1.12  (0.95; 1.32) | 0.68 |
| *Males* | **Low**  **(T1)** | **Moderate**  **(T2)** | **High**  **(T3)** | ***P_trend_*** | **Low**  **(T1)** | **Moderate**  **(T2)** | **High**  **(T3)** | ***P_trend_*** | **Low**  **(T1)** | **Moderate**  **(T2)** | **High**  **(T3)** | ***P_trend_*** |
| Dietary sugar (g/d)^a^ | 47  (41; 50) | 62  (52; 68) | 79  (71; 89) |  | 48  (38; 55) | 61  (55; 72) | 76  (67; 87) |  | 58  (46; 69) | 73  (62; 90) | 99  (86; 114) |  |
| **Inflammatory score** | | | | | | | | | | | | |
| Model A | 0.98  (0.82; 1.18) | 0.90  (0.75; 1.07) | 1.08  (0.90;1.30) | 0.53 | 1.00  (0.84; 1.21) | 0.95  (0.79; 1.14) | 0.99  (0.83; 1.18) | 0.57 | 0.93  (0.78; 1.12) | 0.98  (0.82; 1.17) | 1.03  (0.87; 1.24) | 0.42 |
| Model B | 0.96  (0.80; 1.15) | 0.95  (0.80; 1.12) | 1.05  (0.88;1.25) | 0.54 | 0.99  (0.83; 1.18) | 1.00  (0.84; 1.19) | 0.96  (0.81; 1.14) | 0.64 | 0.90  (0.75; 1.09) | 1.05  (0.89; 1.24) | 0.99  (0.83; 1.18) | 0.43 |
| Model C  (conditional) | 0.95  (0.80; 1.12) | 0.98  (0.84; 1.15) | 1.01  (0.85;1.19) | 0.68 | 0.96  (0.81; 1.12) | 1.02  (0.87; 1.20) | 0.96  (0.82; 1.12) | 0.66 | 0.88  (0.74; 1.04) | 1.10  (0.94; 1.28) | 0.96  (0.82; 1.13) | 0.37 |

Table S1*:* Sex-stratified prospective associations of total dietary fructose, sucrose and glucose intake during adolescence with the inflammatory score in early adulthood (dietary fructose, sucrose and glucose and inflammatory score: n=253 (122 males, 131 females).

Values are adjusted least-squares means (95% CIs) unless otherwise indicated. Linear trends (P_trend_) were obtained in sex-stratified linear regression models with the transformed and energy-adjusted predictors dietary fructose, sucrose, and glucose adolescent intakes as continuous variables. Model A adjusted for adult age at time of blood draw. Model B, with the outcome inflammatory score adjusted for full breastfeeding > 4 months, birth weight, gestational weight gain, paternal education, parental overweight, smoking in the household, pubertal percent body fat, and flavonoid intake. Model C, the conditional model, additionally adjusted for adult percent body fat for all predictors and outcomes. Transformations of variables for analysis: log_e_ for inflammatory score, dietary sucrose and glucose; square root for dietary fructose. ^a^Values are unadjusted medians (25th, 75th percentile). Fructose intake is defined to be free fructose plus 50% of sucrose. Glucose intake is defined to be free glucose plus 50% of sucrose.

|  | ***Tertiles of Total Sugar Intake*** | | | | ***Tertiles of Added Sugar Intake*** | | | | ***Tertiles of Free Sugar Intake*** | | | |
| --- | --- | --- | --- | --- | --- | --- | --- | --- | --- | --- | --- | --- |
| *Females* | **Low**  **(T1)** | **Moderate**  **(T2)** | **High**  **(T3)** | ***P_trend_*** | **Low**  **(T1)** | **Moderate**  **(T2)** | **High**  **(T3)** | ***P_trend_*** | **Low**  **(T1)** | **Moderate**  **(T2)** | **High**  **(T3)** | ***P_trend_*** |
| Dietary sugar (g/d)^a^ | 88  (79; 105) | 113  (103; 129) | 143  (128; 160) |  | 39  (33; 47) | 61  (50; 73) | 78  (70; 95) |  | 52  (43; 60) | 76  (70; 86) | 103  (98; 121) |  |
| **Inflammatory score** | | | | | | | | | | | | |
| Model A | 1.08  (0.90; 1.30) | 0.94  (0.78; 1.12) | 1.03  (0.87; 1.13) | 0.73 | 1.06  (0.89; 1.26) | 0.89  (0.75; 1.06) | 1.11  (0.93; 1.33) | 0.94 | 1.13  (0.94; 1.35) | 0.95  (0.79; 1.14) | 0.98  (0.82; 1.16) | 0.38 |
| Model B | 1.08  (0.90; 1.30) | 0.91  (0.77; 1.09) | 1.05  (0.88; 1.26) | 0.88 | 1.05  (0.85; 1.29) | 0.91  (0.77; 1.08) | 1.10  (0.90; 1.36) | 0.74 | 1.09  (0.91; 1.32) | 0.98  (0.82; 1.18) | 0.97  (0.81; 1.16) | 0.31 |
| Model C  (conditional) | 1.06  (0.90; 1.24) | 0.91  (0.78; 1.06) | 1.09  (0.93; 1.27) | 0.84 | 1.08  (0.91; 1.29) | 0.89  (0.77; 1.03) | 1.11  (0.92; 1.32) | 0.71 | 1.08  (0.92; 1.27) | 1.01  (0.86; 1.18) | 0.97  (0.84; 1.13) | 0.38 |
| *Males* | **Low**  **(T1)** | **Moderate**  **(T2)** | **High**  **(T3)** | ***P_trend_*** | **Low**  **(T1)** | **Moderate**  **(T2)** | **High**  **(T3)** | ***P_trend_*** | **Low**  **(T1)** | **Moderate**  **(T2)** | **High**  **(T3)** | ***P_trend_*** |
| Dietary sugar (g/d)^a^ | 110  (95, 128) | 148  (135, 172) | 173  (150, 200) |  | 51  (42, 68) | 73  (67, 89) | 102  (86, 125) |  | 66  (55, 77) | 92  (81, 111) | 129  (110, 141) |  |
| **Inflammatory score** | | | | | | | | | | | | |
| Model A | 0.94  (0.78; 1.22) | 0.99  (0.83;1.18) | 1.02  (0.85; 1.23) | 0.61 | 0.93  (0.77; 1.12) | 1.00  (0.84; 1.19) | 1.01  (0.84; 1.20) | 0.25 | 1.00  (0.83; 1.20) | 0.90  (0.75; 1.07) | 1.06  (0.88; 1.27) | 0.32 |
| Model B | 0.92  (0.77; 1.09) | 1.03  (0.87;1.22) | 1.00  (0.84; 1.19) | 0.50 | 0.94  (0.78; 1.14) | 1.03  (0.87; 1.22) | 0.97  (0.81; 1.17) | 0.38 | 0.98  (0.83; 1.17) | 0.94  (0.79; 1.11) | 1.03  (0.87; 1.22) | 0.33 |
| Model C  (conditional) | 0.93  (0.79; 1.09) | 1.05  (0.90;1.23) | 0.96  (0.81; 1.12) | 0.50 | 0.91  (0.76; 1.08) | 1.06  (0.91; 1.23) | 0.96  (0.82; 1.14) | 0.37 | 0.95  (0.81; 1.12) | 1.00  (0.86; 1.18) | 0.98  (0.84; 1.15) | 0.48 |

**Table S2***:* Sex-stratified prospective associations of total dietary sugar, added sugar, and free sugar intake during adolescence with the inflammatory score in early adulthood (n=253 (122 males, 131 females).

Values are adjusted least-squares means (95% CIs) unless otherwise indicated. Linear trends (P_trend_) were obtained in sex-stratified linear regression models with the transformed and energy-adjusted predictors dietary fructose, sucrose, and glucose adolescent intakes as continuous variables. Model A adjusted for adult age at time of blood draw. Model B with the outcome inflammatory score adjusted for full breastfeeding > 4 months, birth weight, gestational weight gain, paternal education, parental overweight, smoking in the household, pubertal percent body fat, and flavonoid intake. Model C, the conditional model, additionally adjusted for adult percent body fat for all predictors and outcomes. Transformations of variables for analysis: log_e_ for inflammatory score total sugar intake; square root for added sugar and free sugar intakes. ^a^Values are unadjusted medians (25th, 75th percentile).

|  | ***Tertiles of Urinary Fructose*** | | | | ***Tertiles of Urinary Sucrose*** | | | | ***Tertiles of Urinary Fructose + Sucrose*** | | | |
| --- | --- | --- | --- | --- | --- | --- | --- | --- | --- | --- | --- | --- |
| *Females* | **Low**  **(T1)** | **Moderate**  **(T2)** | **High**  **(T3)** | ***P_trend_*** | **Low**  **(T1)** | **Moderate**  **(T2)** | **High**  **(T3)** | ***P_trend_*** | **Low**  **(T1)** | **Moderate**  **(T2)** | **High**  **(T3)** | ***P_trend_*** |
| Urinary sugar (g/d)^a^ | 10.1  (7.9, 13.3) | 21.2  (19.0, 24.5) | 38.7  (32.3, 54.8) |  | 12.2  (9.4, 15.3) | 25.1  (21.6, 29.1) | 47.2  (34.9, 62.5) |  | 27.0  (21.7, 34.3) | 46.1  (41.0, 52.7) | 79.4  (67.4, 110.9) |  |
| **Inflammatory score** | | | | | | | | | | | | |
| Model A | 1.16  (0.95; 1.41) | 0.97  (0.80; 1.18) | 0.96  (0.79; 1.17) | 0.55 | 1.10  (0.91; 1.33) | 0.97  (0.79; 1.20) | 1.00  (0.83; 1.22) | 0.63 | 1.10  (0.90; 1.34) | 1.03  (0.84; 1.25) | 0.95  (0.79; 1.16) | 0.81 |
| Model B | 1.10  (0.91; 1.34) | 1.00  (0.83; 1.22) | 0.98  (0.81; 1.19) | 0.73 | 1.07  (0.89; 1.30) | 0.97  (0.79; 1.20) | 1.04  (0.86; 1.25) | 0.94 | 0.94  (0.78; 1.14) | 0.96  (0.80; 1.16) | 1.03  (0.85; 1.25) | 0.53 |
| Model C  (conditional) | 1.13  (0.94; 1.34) | 0.94  (0.80; 1.12) | 1.03  (0.86; 1.22) | 0.54 | 1.05  (0.89; 1.23) | 1.03  (0.87; 1.23) | 1.02  (0.87; 1.20) | 0.92 | 1.02  (0.87; 1.21) | 1.13  (0.96; 1.32) | 0.95  (0.82; 1.12) | 0.85 |
| *Males* | **Low**  **(T1)** | **Moderate**  **(T2)** | **High**  **(T3)** | ***P_trend_*** | **Low**  **(T1)** | **Moderate**  **(T2)** | **High**  **(T3)** | ***P_trend_*** | **Low**  **(T1)** | **Moderate**  **(T2)** | **High**  **(T3)** | ***P_trend_*** |
| Urinary sugar (g/d)^a^ | 12.5  (9.9, 14.2) | 22.3  (18.3,23.2) | 37.8  (32.5, 51.7) |  | 15.6  (12.5, 18.9) | 28.3  (25.7, 33.9) | 49.5  (37.4, 68.4) |  | 31.6  (24.8, 37.1) | 52.0  (44.7, 56.0) | 89.7  (75.7, 117.8) |  |
| **Inflammatory score** | | | | | | | | | | | | |
| Model A | 0.86  (0.71; 1.06) | 1.07  (0.88;1.30) | 0.98  (0.80; 1.20) | 0.82 | 0.92  (0.75; 1.13) | 0.94  (0.77; 1.14) | 1.05  (0.86; 1.29) | 0.15 | 0.95  (0.78; 1.16) | 0.92  (0.75; 1.12) | 1.04  (0.85; 1.27) | 0.14 |
| Model B | 0.87  (0.72; 1.05) | 1.10  (0.91;1.31) | 0.98  (0.82; 1.18) | 0.84 | 0.86  (0.70; 1.05) | 1.02  (0.84; 1.23) | 1.05  (0.87; 1.26) | 0.08 | 1.06  (0.87; 1.28) | 1.04  (0.86; 1.26) | 0.99  (0.82; 1.20) | 0.11 |
| Model C  (conditional) | 0.94  (0.79; 1.15) | 1.04  (0.88;1.22) | 0.95  (0.80; 1.12) | 0.46 | 0.91  (0.76; 1.09) | 1.02  (0.86; 1.21) | 0.99  (0.83; 1.17) | 0.53 | 1.00  (0.84; 1.20) | 0.96  (0.81; 1.14) | 0.95  (0.80; 1.13) | 0.61 |

**Table S3***:*  Sex-stratified prospective associations of urinary fructose, urinary sucrose, and the sum of urinary fructose and sucrose excretion during adolescence with the inflammatory score in early adulthood (n=219 (109 males, 110 females).

Values are adjusted least-squares means (95% CIs) unless otherwise indicated. Linear trends (P_trend_) were obtained in sex-stratified linear regression models with the predictors urinary fructose, urinary sucrose, and sum of urinary fructose and sucrose as continuous variables. Model A adjusted for adult age at time of blood draw. Model B, with the outcome inflammatory score adjusted for paternal education, pubertal percent body fat, gestational weight gain, smoking in the household, full breastfeeding > 4 months, as well as excreted hippuric acid, 24-h creatinine, and 24-h urea. Model C, the conditional model, additionally adjusted for adult percent body fat for all predictors and outcomes. Transformations of variables for analysis: log_e_ for excreted urinary sucrose; square root for excreted urinary fructose; log_e_(log_e_) for sum of excreted fructose and sucrose; square root: inflammatory score. ^a^Values are unadjusted medians (25th, 75th percentile).

**Table S4***:* Sex-stratified prospective associations of three sources of total sugar: Sugar-sweetened beverages (SSB), juice, and sweets/sugar intakes during adolescence with markers of insulin sensitivity (n=254: (124 males, 130 females)) and the inflammatory score in early adulthood (n=253 (122 males, 131 females).

|  | ***Tertiles of SSB*** | | | | ***Tertiles of Juice*** | | | | ***Tertiles of Sweets/Sugar*** | | | |
| --- | --- | --- | --- | --- | --- | --- | --- | --- | --- | --- | --- | --- |
| *Females* | **Low**  **(T1)** | **Moderate**  **(T2)** | **High**  **(T3)** | ***P_trend_*** | **Low**  **(T1)** | **Moderate**  **(T2)** | **High**  **(T3)** | ***P_trend_*** | **Low**  **(T1)** | **Moderate**  **(T2)** | **High**  **(T3)** | ***P_trend_*** |
| Dietary sugar (g/d)^a^ | 9.3  (6.3; 12.0) | 19.9  (17.0; 22.8) | 33.4  (29.3; 24.1) |  | 7.7  (3.5; 10.1) | 17.6  (14.1; 18.8) | 27.3  (23.1; 33.9) |  | 22.7  (20.3; ) | 30.2  (27.9; ) | 42.0  (37.9; ) |  |
| **Insulin (pmol/L)** | | | | | | | | | | | | |
| Model A | 75.0  (66.3; 83.7) | 80.9  (72.2; 89.7) | 72.3  (63.1; 81.4) | 0.41 | 74.7  (65.6; 83.8) | 79.1  (70.1; 88.1) | 74.5  (65.7; 83.3) | 0.42 | 76.6  (67.5; 85.7) | 75.0  (66.1; 83.9) | 76.8  (68.1; 85.4) | 0.92 |
| Model B | 71.0  (61.8; 80.3) | 81.0  (72.3; 89.8) | 73.5  (63.8; 83.2) | 0.79 | 76.1  (67.1; 85.2) | 77.0  (68.0; 86.1) | 72.5  (63.6; 81.4) | 0.20 | 74.2  (65.0; 83.3) | 73.0  (64.6; 82.1) | 78.2  (69.4; 86.7) | 0.54 |
| Model C  (conditional) | 72.7  (63.7; 81.8) | 80.2  (71.7; 88.7) | 72.9  (63.5; 82.3) | 0.66 | 76.3  (67.5; 85.0) | 76.2  (67.4; 85.0) | 73.5  (64.8; 82.2) | 0.34 | 74.9  (66.0; 83.8) | 74.1  (65.9; 83.0) | 76.8  (68.3; 85.1) | 0.60 |
| **HOMA2-%S** | | | | | | | | | | | | |
| Model A | 81.3  (73.9; 88.7) | 75.7  (68.2; 83.1) | 82.4  (74.6; 90.2) | 0.45 | 80.6  (72.9; 88.3) | 75.1  (67.5; 82.7) | 83.5  (76.1; 91.0) | 0.40 | 79.8  (72.0; 87.5) | 81.2  (73.6; 88.8) | 78.3  (71.0; 85.7) | 0.97 |
| Model B | 85.7  (78.0; 93.4) | 75.4  (68.0; 82.6) | 80.7  (72.7; 89.0) | 0.85 | 79.2  (71.7; 86.8) | 77.4  (69.9; 84.9) | 85.2  (77.8; 92.6) | 0.20 | 82.1  (74.5; 89.7) | 83.0  (75.6; 90.2) | 77.1  (70.2; 84.3) | 0.51 |
| Model C  (conditional) | 84.6  (76.9; 92.2) | 75.9  (68.7; 83.1) | 81.2  (73.2; 89.2) | 0.70 | 79.1  (71.7; 86.5) | 78.0  (70.5; 85.4) | 84.5  (77.2; 91.9) | 0.35 | 81.6  (74.1; 89.1) | 82.2  (74.9; 89.4) | 78.1  (71.1; 85.0) | 0.57 |
| **Inflammatory score** | | | | | | | | | | | | |
| Model A | 0.95  (0.80; 1.14) | 1.06  (0.89; 1.26) | 1.03  (0.86; 1.24) | 0.21 | 1.04  (0.87; 1.25) | 1.13  (0.95; 1.35) | 0.89  (0.75; 1.06) | 0.13 | 1.17  (0.97; 1.40) | 0.93  (0.78; 1.11) | 0.97  (0.82; 1.16) | 0.18 |
| Model B | 0.88  (0.73; 1.07) | 0.96  (0.79; 1.16) | 1.09  (0.88; 1.35) | 0.02 | 0.98  (0.81; 1.19) | 1.03  (0.85; 1.24) | 0.91  (0.75; 1.10) | 0.35 | 1.10  (0.89; 1.36) | 0.96  (0.80; 1.14) | 0.90  (0.75; 1.08) | 0.06 |
| Model C  (conditional) | 0.93  (0.79; 1.09) | 0.96  (0.82; 1.14) | 1.04  (0.87; 1.25) | 0.05 | 1.00  (0.85; 1.18) | 0.98  (0.83; 1.15) | 0.95  (0.81; 1.11) | 0.59 | 1.10  (0.92; 1.32) | 1.00  (0.86; 1.16) | 0.87  (0.75; 1.00) | 0.04 |
| *Males* | **Low**  **(T1)** | **Moderate**  **(T2)** | **High**  **(T3)** | ***P_trend_*** | **Low**  **(T1)** | **Moderate**  **(T2)** | **High**  **(T3)** | ***P_trend_*** | **Low**  **(T1)** | **Moderate**  **(T2)** | **High**  **(T3)** | ***P_trend_*** |
| Dietary sugar (g/d)^a^ | 8.6  (5.8; 12.5) | 20.2  (18.6; 22.6) | 32.3  (28.1; 38.3) |  | 9.1  (6.2; 12.3) | 17.7  (16.8; 19.3) | 30.1  (23.4; 33.9) |  | 22.6  (18.4; 23.8) | 30.7  (27.6; 33.1) | 43.1  (38.3; 46.6) |  |
| **Insulin (pmol/L)** | | | | | | | | | | | | |
| Model A | 70.8  (59.5; 82.1) | 77.9  (66.8; 88.9) | 68.3  (57.1; 79.6) | 0.88 | 68.5  (56.5; 80.4) | 75.1  (64.1; 86.1) | 73.3  (61.7; 84.9) | 0.99 | 65.2  (54.1; 76.4) | 78.8  (67.7; 89.9) | 72.9  (61.6; 84.2) | 0.99 |
| Model B | 70.6  (58.9; 82.3) | 78.4  (67.2; 89.6) | 69.4  (58.3; 80.4) | 0.78 | 69.0  (57.2; 80.8) | 75.7  (64.7; 86.7) | 73.4  (61.3; 85.4) | 0.94 | 65.3  (53.4; 76.0) | 79.6  (68.1; 90.7) | 73.0  (61.9; 84.2) | 0.91 |
| Model C  (conditional) | 70.8  (59.1; 82.5) | 78.2  (66.9; 89.4) | 69.2  (58.1; 80.4) | 0.85 | 68.9  (57.1; 80.7) | 75.7  (64.7; 86.7) | 73.3  (61.3; 85.3) | 0.99 | 65.2  (53.8; 76.6) | 79.5  (68.2; 90.8) | 72.4  (61.1; 83.6) | 0.65 |
| **HOMA2-%S** | | | | | | | | | | | | |
| Model A | 86.3  (76.0; 96.6) | 82.0  (71.9; 92.1) | 87.1  (76.9; 97.4) | 0.93 | 88.9  (78.1; 99.7) | 84.3  (74.4; 94.3) | 82.2  (71.7; 92.6) | 0.96 | 91.4  (81.2; 101.5) | 81.5  (71.4; 91.6) | 82.5  (72.2; 92.8) | 0.95 |
| Model B | 87.4  (76.7; 98.2) | 83.0  (72.7; 93.3) | 85.9  (75.7; 96.1) | 0.72 | 88.5  (77.7; 99.3) | 84.8  (74.7; 94.9) | 82.9  (71.9; 93.9) | 0.89 | 91.9  (81.5; 102.3) | 80.9  (70.5; 91.2) | 83.7  (73.5; 94.2) | 0.97 |
| Model C  (conditional) | 87.0  (76.3; 97.6) | 83.6  (73.4; 93.8) | 86.2  (76.1; 96.3) | 0.79 | 88.7  (78.0; 99.4) | 84.8  (74.9; 94.8) | 83.0  (72.1; 93.9) | 0.94 | 90.9  (80.6; 101.3) | 80.7  (70.4; 91.0) | 85.2  (74.9; 95.6) | 0.70 |
| **Inflammatory score** | | | | | | | | | | | | |
| Model A | 0.92  (0.76; 1.11) | 1.01  (0.85; 1.21) | 1.00  (0.84; 1.21) | 0.61 | 1.06  (0.88; 1.28) | 0.89  (0.75; 1.06) | 1.01  (0.83; 1.22) | 0.45 | 0.88  (0.73; 1.06) | 1.03  (0.86; 1.23) | 1.04  (0.87; 1.25) | 0.24 |
| Model B | 1.01  (0.81; 1.27) | 1.02  (0.83; 1.25) | 0.90  (0.74; 1.10) | 0.62 | 0.96  (0.78; 1.18) | 0.84  (0.70; 1.02) | 1.13  (0.92; 1.39) | 0.83 | 0.86  (0.71; 1.04) | 1.03  (0.84; 1.25) | 1.03  (0.84; 1.25) | 0.27 |
| Model C  (conditional) | 1.02  (0.83; 1.25) | 0.98  (0.81; 1.19) | 0.92  (0.77; 1.10) | 0.66 | 0.97  (0.81; 1.17) | 0.85  (0.72; 1.02) | 1.10  (0.90; 1.33) | 0.30 | 0.89  (0.75; 1.06) | 1.04  (0.87; 1.25) | 0.97  (0.81; 1.17) | 0.85 |

Values are adjusted least-squares means (95% CIs) unless otherwise indicated. Linear trends (P_trend_) were obtained in sex-stratified linear regression models with the predictors sugar-sweetened beverages (SSB), juice, and sweets/sugar intakes as continuous variables. Model A adjusted for adult age at time of blood draw. Model B, with the outcome inflammatory score adjusted for paternal education, pubertal percent body fat, gestational weight gain, smoking in the household, full breastfeeding > 4 months, as well as excreted hippuric acid, 24-h creatinine, and 24-h urea. Model B, with outcomes HOMA2-%S and fasting insulin, additionally adjusted for paternal education, birth weight, gestational weight gain, smoking in the household, parental overweight and pubertal percent body fat. Model C, the conditional model, additionally adjusted for adult percent body fat for all predictors and outcomes. Transformations of variables for analysis: SSB, juice used square root transformation; HOMA2-%S and fasting insulin used log_e_. ^a^Values are unadjusted medians (25th, 75th percentile)

**Table S5***:* Sex-stratified regression results from substitution models that replace respective sugars with non-sugar carbohydrates. Estimates reflect the substation of various sugar types with non-sugar carbohydrates in models B and C with the inflammatory score in early adulthood (n=253 (122 males, 131 females).

| **Inflammatory score and dietary sugars** | | | | | |
| --- | --- | --- | --- | --- | --- |
| **Substitution models:**  **Replacing respective sugar with non-sugar carbohydrates** | | Estimate | | P-value | |
|  |  | Male | Female | Male | Female |
| **Added Sugar** | B:  C: | 0.018  0.321 | -0.363  -0.044 | 0.68  0.42 | 0.50  0.38 |
| **Free Sugar** | B:  C: | 0.022  0.034 | -0.055  -0.044 | 0.58  0.34 | 0.23  0.26 |
| **Total Sugar** | B:  C: | 0.000  0.014 | -0.003  0.001 | 0.97  0.71 | 0.95  0.99 |
| **Dietary Fructose** | B:  C: | 0.013  0.013 | -0.071  -0.058 | 0.80  0.77 | 0.20  0.23 |
| **Dietary Glucose** | B:  C: | 0.014  0.036 | -0.099  -0.07 | 0.79  0.47 | 0.098  0.17 |
| **Dietary Sucrose** | B:  C: | 0.021  0.039 | -0.047  -0.050 | 0.63  0.33 | 0.38  0.28 |
| **SSB** | B:  C: | -0.011  -0.001 | 0.018  0.021 | 0.75  0.97 | 0.65  0.53 |
| **Juice** | B:  C: | -0.016  -0.001 | -0.038  -0.02 | 0.66  0.85 | 0.27  0.47 |
| **Sweets/Sugar** | B:  C: | 0.032  0.017 | -0.068  -0.67 | 0.32  0.56 | 0.08  0.04 |

Estimate values were obtained in sex-stratified linear regression models with sugar predictors as continuous variables. Model B, with the outcome pro-inflammatory score adjusted for full breastfeeding > 4 months, birth weight, gestational weight gain, paternal education, parental overweight, smoking in the household, pubertal percent body fat, and flavonoid intake. The conditional Model C additionally adjusted for adult percent body fat for all predictors and outcomes. Additionally, each model included total energy intake, dietary protein, dietary fat, the respective sugar & sugar rest variables i.e. total sugar – respective sugar. These energy-bearing variables were held constant and non-sugar carbohydrates were left out of the model in order to simulate the substitution of each respective sugar for non-sugar carbohydrates. Total sugars from SSB, juice and sweets/sugar as sources are used.

**Table S6***:* Sex-stratified regression results from substitution models that replace respective sugars with non-sugar carbohydrates. Estimates reflect the substation of various sugar types with non-sugar carbohydrates in models B and C with markers of insulin sensitivity (n=254: (124 males, 130 females)) in early adulthood.

| **Substitution models:**  **Replacing respective sugar with non-sugar carbohydrates** | | **Insulin Sensitivity** | | | | **Fasting Insulin** | | | |
| --- | --- | --- | --- | --- | --- | --- | --- | --- | --- |
|  |  | Estimate | | P-value | | Estimate | | P-value | |
|  |  | Male | Female | Male | Female | Male | Female | Male | Female |
| **Added Sugar** | B:  C: | 0.011  0.006 | 0.006  0.012 | 0.90  0.95 | 0.94  0.87 | -0.006  -0.000 | -0.208  -0.026 | 0.95  0.99 | 0.80  0.73 |
| **Free Sugar** | B:  C: | 0.009  0.003 | 0.004  -0.000 | 0.91  0.97 | 0.95  0.98 | -0.006  0.000 | -0.015  -0.010 | 0.94  0.99 | 0.83  0.89 |
| **Total Sugar** | B:  C: | 0.023  0.013 | 0.032  0.027 | 0.79  0.88 | 0.66  0.71 | -0.013  -0.003 | -0.042  -0.036 | 0.88  0.97 | 0.57  0.62 |
| **Dietary Fructose** | B:  C: | 0.040  0.048 | 0.090  0.072 | 0.61  0.63 | 0.29  0.38 | -0.043  -0.039 | -0.093  -0.077 | 0.67  0.69 | 0.27  0.36 |
| **Dietary Glucose** | B:  C: | 0.012  -0.003 | 0.002  -0.014 | 0.92  0.98 | 0.98  0.87 | -0.016  -0.001 | -0.001  0.015 | 0.89  0.99 | 0.99  0.87 |
| **Dietary Sucrose** | B:  C: | 0.066  0.059 | 0.029  0.037 | 0.45  0.50 | 0.71  0.63 | -0.061  -0.053 | -0.043  -0.051 | 0.49  0.54 | 0.59  0.51 |
| **SSB** | B:  C: | 0.009  0.007 | 0.015  0.013 | 0.90  0.92 | 0.86  0.82 | -0.006  -0.004 | -0.019  -0.022 | 0.93  0.95 | 0.74  0.71 |
| **Juice** | B:  C: | 0.028  0.020 | 0.05  0.036 | 0.69  0.77 | 0.32  0.48 | -0.021  -0.013 | -0.058  -0.042 | 0.76  0.85 | 0.27  0.42 |
| **Sweets/Sugar** | B:  C: | 0.020  0.031 | -0.049  -0.046 | 0.76  0.65 | 0.37  0.40 | -0.020  -0.032 | 0.044  0.040 | 0.77  0.64 | 0.44  0.47 |

Estimate values were obtained in sex-stratified linear regression models with sugar predictors as continuous variables. Model B, with outcomes insulin sensitivity and fasting insulin, additionally adjusted for paternal education, pubertal percent body fat and gestational weight gain. The conditional Model C additionally adjusted for adult percent body fat for all predictors and outcomes. Additionally, each model included total energy intake, dietary protein, dietary fat, the respective sugar & sugar rest variables i.e. total sugar – respective sugar. These energy-bearing variables were held constant and non-sugar carbohydrates were left out of the model in order to simulate the substitution of each respective sugar for non-sugar carbohydrates. Total sugars from SSB, juice and sweets/sugar as sources are used.
